# Inositol phosphates: small molecules with a BIG impact on maize embryo development

**DOI:** 10.1093/plphys/kiag059

**Published:** 2026-02-11

**Authors:** Catherine P Freed, Erin Cullen

**Affiliations:** Department of Biochemistry, University of Wisconsin-Madison, Madison, WI 53706, United States; Plant Physiology, American Society of Plant Biologists; Plant Physiology, American Society of Plant Biologists

Plants utilize complex signaling networks to coordinate nutrient allocation and storage across tissues. In maize and other cereal crops, traits such as embryo and endosperm size are key determinants of oil, protein, starch, and nutrient storage. Understanding the molecular foundations of embryo size and nutrient storage is essential for developing nutrient rich crops.

Inositol phosphates (InsPs) are a diverse group of signaling molecules that, depending on their phosphorylation pattern, can convey unique chemical information (Gillaspy 2011). InsPs are important for plant development, phosphate sensing, hormone signaling, and response to abiotic and biotic stress (Ghosh et al. 2025). Consisting of a 6-carbon inositol ring scaffold, InsPs are phosphorylated by a specific set of enzymes in 2 interconnected pathways that are lipid dependent and lipid independent (Williams et al. 2015; Shears 2018). Although many key enzymes involved in InsP signaling have been identified in plants, there are still many mechanistic unknowns surrounding how specific InsPs contribute to plant development and stress responses. Understanding how InsPs impact specific developmental processes is critical given they are attractive targets for enhancing nutrient use efficiency and stress resilience in crops (Freed et al. 2020),

A recent study in *Plant Physiology* by Suzuki et al. (2026) investigates the link between InsP signaling and maize embryo development. To determine the genetic controllers of maize embryo size, the authors identified 4 maize mutants with enlarged embryos from a previous screen (McCarty et al. 2005). Interestingly, all 4 identified mutants had mutations in the InsP signaling pathway: *big embryo2* (*bige2*) and *big embryo3* (*bige3*), deficient in lipid independent InsP kinases; *big embryo4* (*bige4*), impaired in a lipid dependent InsP kinase; and *low phytic acid 1* (*lpa1*), lacking a tonoplast InsP_6_ transporter (Fig. 1). Including mutants involved in different aspects of InsP signaling allowed the authors to study how InsP enzymes spanning multiple parts of the signaling pathway contribute to embryo development.

**Figure 1:**
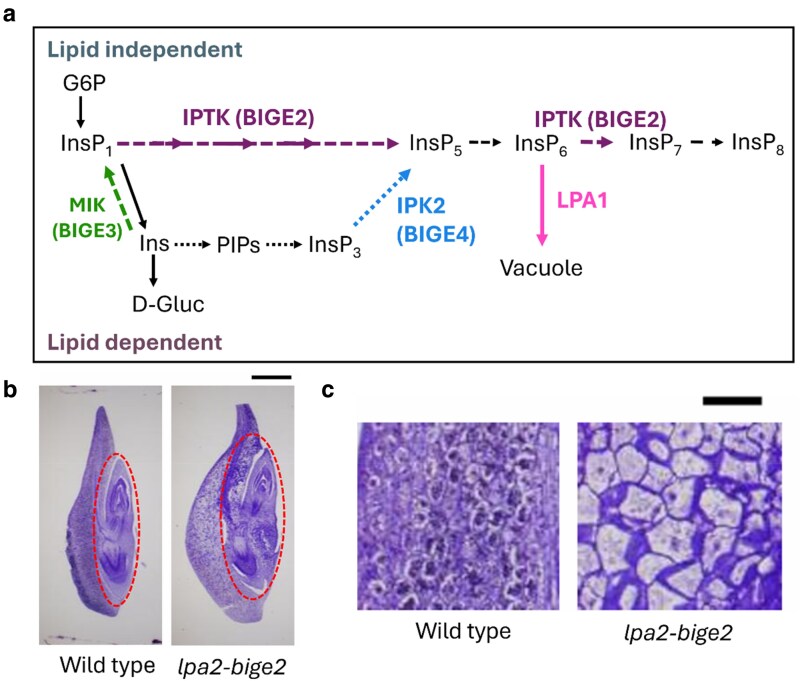
*BIG EMBRYO* mutations in maize affect both lipid-dependent and -independent inositol phosphate signaling pathways, increasing embryo size at the expense of the endosperm (adapted from Figures 2 and 3 in Suzuki et al. 2026). **(a)** Schematic indicating the roles of *BIG EMBRYO* and *LPA1* genes in the lipid-dependent and -independent inositol phosphate signaling pathways. Proteins of interest and their impact on the pathway are represented by different colors (BIGE2: purple, BIGE3: green, BIGE4: blue, and LPA1: pink). Dotted arrows represent lipid-dependent pathway reactions, and dashed arrows represent lipid-independent pathway reactions. **(b)** The *bige2* mutant has an enlarged scutellum compared to WT, indicated by a red dashed line. **(c)** Larger cell size underlies the enlarged scutellum in *bige2*. Sections from the apical scutellum reveals enlarged cells compared to WT.

All mutants displayed alterations in scutellum growth based on changes in cell size and/or number at the expense of the endosperm (Fig. 1). Notably, *bige* mutants showed higher accumulation of anthocyanins in certain areas of the kernels as well as distinct changes in germination frequency compared to wild-type (WT) kernels. Of these, *bige2* mutants showed the most distinctive phenotypes in size and coloration compared to the other mutants. Together, this suggests that while these genes have overlapping roles in altering embryo size, they also have distinct roles in maize development.

To dissect changes in increased embryo size, the authors used histological analyses to quantify cell size in *bige2* and *bige4* embryos at later stages in kernel development. Although both mutants had an enlarged scutellum, the *bige2* scutellum was larger due to increased cell size, whereas the *bige4* scutellum had an increased number of cells. The authors found that the increase in cell size may be due to an increase in ploidy. Upon further inspection of the embryos at apical, middle, and basal locations, the authors found that *bige2* had significantly larger cells in the apical region, while in *bige4* cell size was not largely altered at any location. Together, these results establish that alterations in specific InsP signaling genes impact cell size and number in specific regions in the embryo.

The authors also extended their histological analysis to query scutellum size in all 4 mutants at the onset of embryo development. Both *bige2* and *lpa1* scutellum were significantly larger in the apical region and *bige3* scutellum cells were slightly larger compared to WT. In *bige4*, some areas of the scutellum had larger cells compared to WT, whereas in the apical region, while the cells were not significantly larger in size, they were higher in number. Together, these results establish that alterations in specific InsP signaling genes impact cell size and number in specific regions in the embryo.

Suzuki et al. (2026) also compared the transcriptomes of WT and *bige* embryos. Of these, *bige2* mutant embryos showed the most changes with ∼4,600 differentially expressed genes (DEGs), consistent with their stronger kernel phenotypes, while *bige3* mutants had under 100 DEGs and *bige4* mutants had ∼2,100 DEGs. These 2 mutants shared lipid accumulation and histone DEGs, suggesting that these genes are uniquely regulated by the lipid independent InsP kinases. A small subset of 13 genes was shared between all 3 *bige* mutants with roles in RNA biogenesis and ion homeostasis. Interestingly, over one-half of the upregulated DEGs were shared between *bige2* and *bige4* embryos with roles in protein and ribosomal translation, phosphate and lipid metabolism, DNA replication and repair, and the cell cycle. These data suggests that the InsP kinases from both pathway impact cell cycle regulation in mutant embryos.

The authors also quantified levels of genes previously identified to regulate embryo size in maize, such as *CYP78A* and *BIGE1-LIKE MATE* transporter genes. Multiple *CYP78A* genes were downregulated in *bige2* and *bige4* mutants, whereas *BIGE1-LIKE MATE* genes were upregulated in *bige2*, consistent with past literature (Suzuki et al. 2015). Mutant embryo transcriptome analyses also revealed anthocyanin synthesis genes in the *bige* mutants that were consistent with the observed kernel phenotypes.

This work uniquely establishes a role for InsPs in regulating embryo size and cell development in maize. Knocking out genes that span multiple parts of the InsP signaling pathway highlights both overlapping and distinct roles for InsP enzymes in regulating embryo development and the cell cycle in maize. This research is valuable given ongoing efforts that target InsPs to enhance crop resilience and nutrient use (Raboy 2007; Freed et al. 2020). Given the importance of InsPs in phosphate sensing and hormone signaling pathways such as auxin and jasmonic acid, future efforts exploring *bige* and *lpa1* maize mutant defense response to abiotic and biotic stress would be informative. Future studies should also quantify and examine how specific InsP levels contribute to *bige* and *lpa1* maize phenotypes.

## Data Availability

No new data were generated or analyzed in support of this research.
